# Facile Fabrication of Polymeric Microfluidic Channel Based on Fibrous Mold Base Using Near-Field Electrospinning

**DOI:** 10.3390/polym18192444

**Published:** 2026-10-07

**Authors:** Chan Hee Kim, Hae Woon Choi, Jong Hyun Kim

**Affiliations:** Department of Mechanical Engineering, Keimyung University, 1095 Dalgubeol-daero, Daegu 42601, Republic of Korea; jimi67676@gmail.com (C.H.K.); hwchoi@kmu.ac.kr (H.W.C.)

**Keywords:** microfluidic, channel, fiber, electrospinning, patterning

## Abstract

Microfluidic channels underpin lab-on-a-chip and analysis systems, yet their fabrication still often relies on cleanroom-intensive, photomask-based processes that limit rapid prototyping and user accessibility. Here, we present a facile fabrication strategy that uses near-field electrospinning (NFES) of polyethylene oxide (PEO) fibers as a sacrificial mold base, followed by polydimethylsiloxane (PDMS) replication and simple glass–PDMS assembly, to realize closed, capillary-driven microchannels without conventional master molds. NFES enables direct writing of straight fiber and reservoir-dot patterns on substrates, and systematic control of the applied electric field tunes fiber widths in the tens-of-micrometers range, yielding semi-elliptical channel cross-sections that are faithfully transferred into PDMS. The fiber width was controlled to approximately 34–100 μm by adjusting the voltage of the NFES to 1.4–1.9 kV. Theoretical calculations and numerical analysis showed that as the channel width decreased, the curvature of the liquid interface and the capillary driving pressure increased, leading to an increase in the flow velocity and capillary filling. Through the study, it was learned that the channel shape is a key design variable for the non-powered flow control. Experiments with aqueous solutions confirm spontaneous, pump-free capillary transport in the fabricated devices and demonstrate enhanced acceleration and reversal of flow by light-induced modulation. Therefore, the proposed NFES-based fibrous mold-based approach thus offers a simple, affordable, and facile route to microfluidic channels supporting flow control, making it well suited for rapid prototyping and iterative optimization of capillary microfluidic platforms for biosensing, sample delivery, and lab-on-a-chip applications.

## 1. Introduction

Microfluidic channels constitute the fundamental structures of lab-on-a-chip, biosensing, sample delivery, and fluid analysis platforms, providing precise solutions for the controlled movement, mixing, reaction, and analysis of minute amounts of liquid [1,2,3]. These applications require fabrication techniques that enable precise channel geometry, rapid prototyping, and design modification. Various methods have been developed to achieve precise fabrication, which is a key requirement for microchannels. The various fabrication methods developed have their own advantages and disadvantages regarding process applicability.

As shown in Table 1, conventional fabrication routes—such as photolithography, sacrificial template, and 3D printing techniques—have substantially advanced microfluidic technology but remain constrained by cleanroom dependence, expensive machines, limited material compatibility, and user restrictions that hinder rapid prototyping [4,5]. In particular, in conventional polydimethylsiloxane (PDMS) soft lithography, a master mold is typically fabricated using a photomask, and then the channel geometry is replicated onto PDMS. Therefore, changing the channel design entails fabricating a new master mold, resulting in high manufacturing costs and processing time. These limitations have led to an increasing demand for alternative manufacturing strategies capable of generating microscale sacrificial templates or direct channel structures with reduced process complexity and lower manufacturing costs [6].

**Figure 1:**
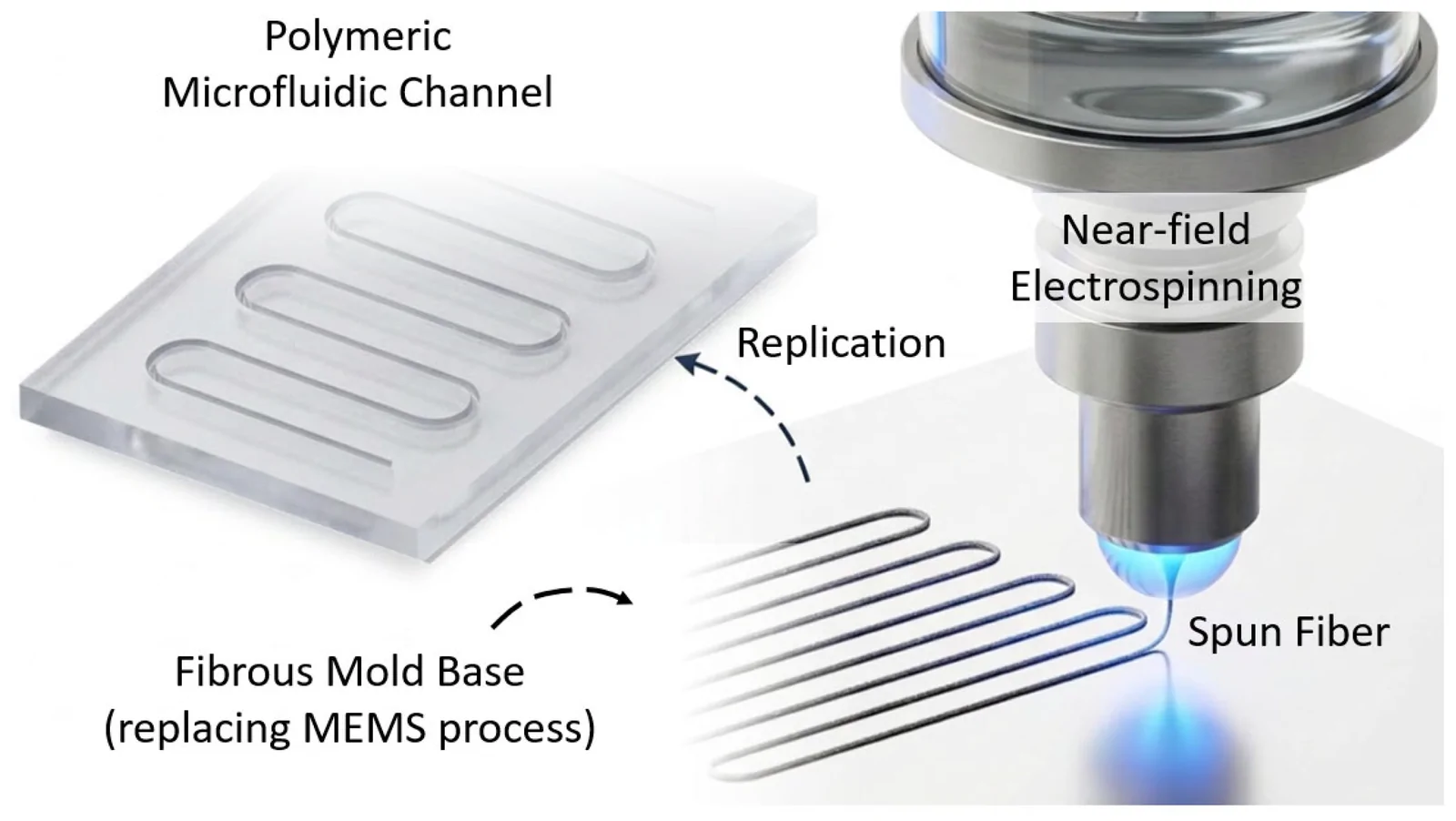
Concept of facile fabrication of polymeric microfluidic channel based on fibrous mold base. The microfiber pattern is prepared through near-field electrospinning. Liquid polymer is cast over the spun fiber mold to form a replicated polymeric microfluidic channel. The method replaces traditional microfabrication processes.

Recent progress in additive manufacturing has broadened the design space of microfluidic devices via material extrusion, photopolymerization, imprint, and related approaches [7,8,9]. While general additive manufacturing allows for the direct fabrication of structures based on digital models, which is advantageous for realizing complex geometries and design modifications, minimum geometry resolution and product quality vary depending on the printing method, equipment, and materials used. Among these, Fused Filament Fabrication (FFL) offers a low-cost method but often fails to realize the smooth and dimensionally precise microchannel surfaces required for predictable microflow behavior. Vat Photopolymerization (VPP) can achieve higher resolution and finer geometries, but generally has disadvantages such as expensive equipment and complex process control [9]. Electro-assisted printing technology has been developed to create precise geometries. Electrohydrodynamic (EHD) printing enables rapid fabrication, but its resolution is limited to hundreds of micrometers. Moreover, the sacrificial template method forms a closed channel by placing a removable material inside the structure and then removing it [10]. However, while these methods enable the realization of complex shapes, they show clear limitations in terms of resolution and process simplicity. Referring to the conceptual image shown in Figure 1, this study proposes a facile and reliable fabrication method that utilizes polymeric fibers patterned by near-field electrospinning (NFES) as a surface-embossing mold base for PDMS replication. NFES is highly attractive for forming channel mold bases with diverse and precisely controlled layouts because it can directly deposit polymer fibers in programmable patterns with linewidths ranging from nanometers to micrometers at short tip–collector distances. After simply fabricating a fiber pattern, we were able to successfully fabricate channels through a process involving silane surface treatment, PDMS replication, UV–ozone treatment, and bonding. By exploiting these easily patterned fibers as transferable sacrificial templates, NFES offers a straightforward route to microfluidic channels that circumvents multiple steps of conventional photolithography-based master mold fabrication process [11]. To diversify channel sizes, the width of the fibers was controlled within the range of approximately 34–100 μm by adjusting the applied voltage, and the formed fiber mold bases were reused for high-volume works. Advanced design and manufacturing were achieved through microscale and computer simulation analysis. The central hypothesis is that sacrificial fiber networks produced by NFES can define uniform, linear microchannel geometries in fewer steps than conventional cleanroom processes while preserving the optical transparency, accessibility, and versatility of PDMS-based microfluidic devices, and successful channel fabrication is possible with a few dollars for materials and post-processing.

Through these processes, we present that the main contribution of this study lies in utilizing directly patterned fibers as a mold base to accomplish channel formation, PDMS mold base fabrication, and capillary flow control for a quickly integrated fabrication process [11,12,13,14,15,16]. These findings utilize a useful foundation for designing microfluidic systems optimized for applications such as medical devices, sample delivery, and lab-on-a-chip platforms [17].

## 2. Methods and Materials

### 2.1. Materials and Tools

For molding, PDMS (Sylgard184, Sylgard 184A, Dow Corning, USA; supplied by Sewang Hitech, Republic of Korea) was chosen as the mold, because it is not only transparent, but also reliably functions in microfluidic devices. Chlorotrimethylsilane (silane, purity of 98.0%, special grade, SAMCHUN, Republic of Korea, CAS no. 75-77-4) was used for separation between PDMS mold and silicon wafer. An ultraviolet (UV)–ozone cleaner (UVC-150, Omniscience, Republic of Korea) was applied for surface treatment. For NFES, the polymer solution was based on polyethylene oxide (PEO, 400,000 g/mole, Sigma-Aldrich), dissolved with DI water for 24 h using a magnetic stirrer (MS3000, Static 3000 rpm, 12V DC, 6 W) to make 8 wt % PEO solution. PEO dissolved in deionized water was selected, which provides an appropriate viscosity (35 Pa s) for the NFES. A viscometer (ubbelohde type, CANON) was used at room temperature. Under all NFES conditions using the same PEO solution, it was confirmed that the electric field plays a key role in the formation and size of the fibers. The surface tension is in the range of approximately 62 ± 2 mN/m at room temperature [18]. A 24-gauge-sized metal needle (outer diameter at 0.515 mm, NordsonVR, Westlake, OH, USA) was used as the syringe tip. A 4-inch silicon wafer was chosen as the collector. A syringe pump (Legato 110, KD Scientific, USA) was used for automatic water material supply for NFES and contact angle measurement. The needle tip and the collector were connected to a high-voltage direct current supply (NNC-HV30, Nano NC, Republic of Korea). The programmable XY linear stage (a resolution of 1 μm, Aero System, Republic of Korea) translated the collector, and Z linear stage (GTS30V, Res. 0.1 μm, Aero System, Republic of Korea) controlled the distance between the needle and the silicon wafer. Power supply and stages were controlled by the customized graphic user interface with the real-time monitoring system so that the corresponding electrostatic forces can be modified during a single continuous process. The monitoring system includes a microscope and camera. Images were obtained using a high-magnification microscope (OLYMPUS SZ series, Olympus Corporation, Japan; stand: SZ2-ST) and camera (Eakins, 4K, USB type). Fibrous surfaces are visually analyzed by 3D scanning (VK-X1000, Keyence, Itasca, IL, USA).

### 2.2. Key Idea: Near-Field Electrospinning

**Figure 2:**
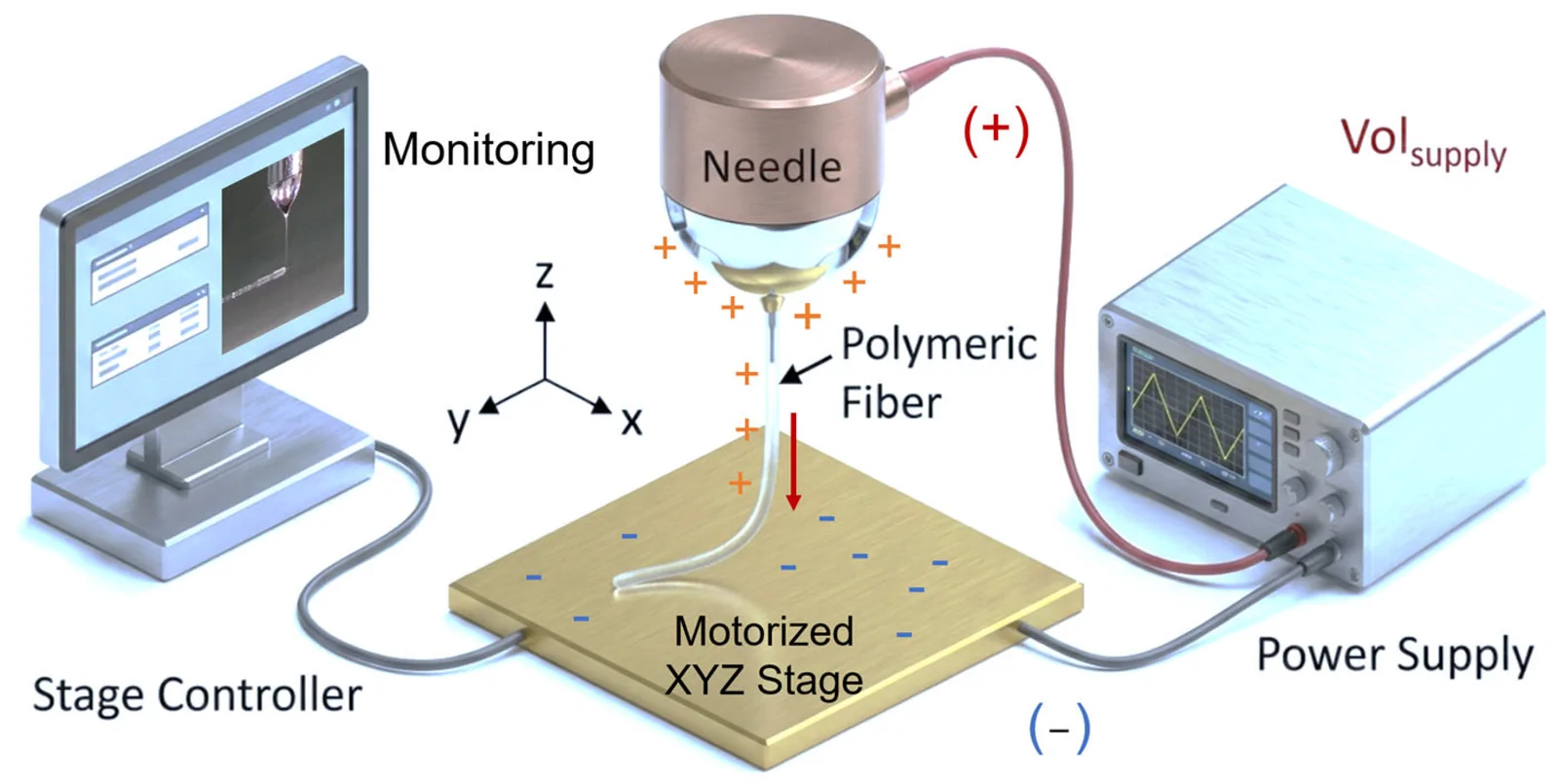
Near-field electrospinning system including monitoring and control units. When the power supply provides electrical potential difference, polymeric fibers ejected from the needle are deposited on the target collector. Fibers can be placed in desired patterns and locations using a programmable stage, and process reliability is achieved through real-time monitoring.

Figure 2 provides an NFES system and principle for fiber patterning. As described above, we propose that NFES is a significant method for making mold bases for channel fabrication [19]. NFES not only produces fibers with nano-to-micro diameters but can also create desired drawings such as straight and curved lines. In particular, NFES can directly form fine-scale line templates for on-demand positions, thereby simplifying the traditional deposition–lithography–etching processes, which is a pre-process for PDMS channel production [20]. The metal needle tip and the silicon wafer are connected to the power supply to provide the electric potential required to generate an EF. The highly doped silicon wafer is positioned on the XY-Z stage to act as a conductive collector [21]. The programmable XY-Z linear stage is used to control the position of the fiber deposition and the distance from the needle tip to the collector. The ejection of the polymer solution, based on droplet size, electric field (EF), viscosity of solution, and tip-to-collector distance (TTCD), is monitored and controlled in real time. Polyethylene oxide (PEO) was selected as the solution because it not only converts into ultrafine fibers due to its appropriate viscosity but also prevents solidification during movement between the syringe tip and the collector by using deionized water as a solvent [11].

While operating the system, viscous polymeric PEO pumped by a microfluidic pump forms spherical droplets due to surface tension [15]. The potential difference provides a force that pulls the droplets downward. In terms of droplet size control, the surface tension of the solution, the hydrophobicity of the needle, and the diameter of the needle tip are dominant. At the beginning of the process, the polymer is controlled by controlling the pump until a droplet of a predetermined size is formed at the nozzle end. Then, once the target size is confirmed through real-time monitoring, the pump is turned off. In other words, during fiber patterning, fibers were ejected using droplets formed at the needle tip without a continuous supply of polymer solution via the pump. For expansion of production, a continuous supply of material using the pump is required. It should be noted that the droplet size must be appropriate to maintain a balance between surface tension and EF forces. The larger the droplet size, the greater the influence of the EF [14].

After droplet formation, a voltage is applied between the metal needle and the conductive collector [22]. As the voltage potential increases, charges accumulate within the polymer toward the collector. Due to the Coulomb attraction of charges, the droplet overcomes the surface tension, and a fiber jet is ejected downward, as can be seen in the central image of the figure. This demonstrates that an EF exceeding the surface tension of the polymer disrupts the equilibrium between the droplet and the needle. As the microfiber flies from the droplet to the collector, it is exposed to EF and gravity, contributing to the formation of the fiber. Finally, the computer-programmable XY stage assists through desired shapes and on-demand fiber deposition. In this study, the TTCD was fixed at 0.6 mm, and the applied voltage was adjusted in increments of 0.1 or 0.2 kV within the range of 1.1–1.9 kV. Among these, voltages of 1.4, 1.5, 1.7, and 1.9 kV were applied for channel width comparison. Additionally, the travel speed of the XY stage for linear fiber patterning was set to 0.7 mm/s, and the experiment was conducted in an environment with a temperature of 78–80 °F and a relative humidity of approximately 20%.

### 2.3. Quick Fabrication

**Figure 3:**
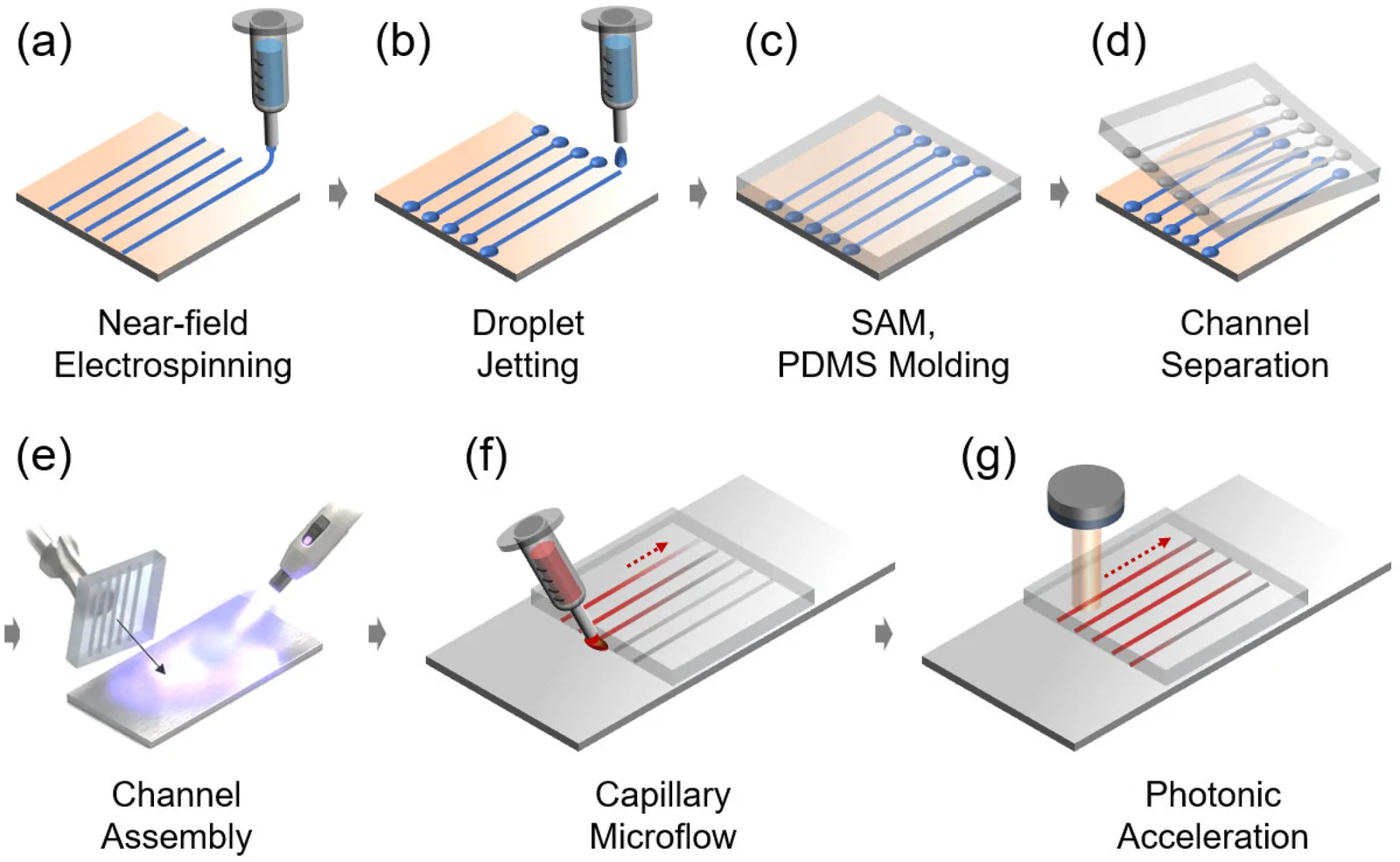
Schematic of fabrication processes. (**a**) Fiber patterning through near-field electrospinning. (**b**) Droplet jetting using excessive EF in the same electrospinning system. (**c**) Hydrophobic surface treatment and molding for easy separation. (**d**) Separation of polymeric channel mold after heat curing. (**e**) Surface cleaning using UV–ozone and assembly with glass slide. (**f**) Capillary force-driven microflow in the fabricated channels. (**g**) Flow control using light-driven acceleration.

Figure 3 illustrates the essential fabrication process of the NFES-based microfluidic channel proposed in this study. The process features a particularly simple fabrication sequence. It is fabricated through a small number of steps, such as fiber fabrication, silane coating for interfacial adhesion management, PDMS molding, and surface cleaning. As a result, a channel capable of microfluidic movement without pumping is functional. Notably, our approach avoids photomask and reduces reliance on master mold preparation, which requires expensive equipment and many steps, making it advantageous for quick fabrication and iterative optimization. Based on this workflow, we present a linear channel prototype to demonstrate the concept of easy fabrication and facile production. A linear channel prototype was constructed to demonstrate simple production and the possibility of repetitive replication.

First, as shown in Figure 3a, a linear fiber pattern can be fabricated on the silicon wafer using the NFES process. The pattern was quickly formed according to the movement path of the XY stage programmed under the previously described NFES conditions. Unlike conventional 3D printing, which forms layered closed channels, this step directly fabricates microscale embossed structures using NFES, which serve as the mold base for manufacturing channel molds. In Figure 3b, dot structures are formed at the ends of the pattern through droplet ejection [23]. When an excessive EF is applied to the NFES system, the droplets detach from the needle and fall to the collector due to gravity [24]. When the accelerated viscous droplets strike the collector surface, a dome-shaped circular structure is formed. This dome structure serves as the inlet and outlet reservoir for the channel mold. Between steps (b) and (c), a hydrophobic self-assembled monolayer (SAM) surface coating is applied to separate the fibers from the PDMS to be fabricated in the next step [25]. The silicon wafer containing the fibers is placed in a vacuum chamber for one hour to perform the silane coating. In total, 10 g of the silane was used for the surface treatment, and the vacuum chamber was maintained at approximately −0.017 MPa. The silane coating reduces the adhesion between the deposited fibers, the wafer, and the PDMS, facilitating the subsequent separation or transfer of the PDMS. In Figure 3c, replication molding is performed using PDMS, a polymeric elastomer. The PDMS is mixed with a curing agent at a ratio of 10:1. A mini vacuum chamber is utilized to remove air. If air remains inside the PDMS, it can interfere with flow and visualization monitoring. Since residual bubbles can cause the shape of the replicated channels to be non-uniform, a degassing process was performed in a vacuum chamber before injecting PDMS into the substrate. About 8 g of the PDMS mixture was injected into the silicon wafer with a diameter of about 100 mm to a thickness of about 1 mm. The PDMS is cured by heating it on a hot plate at 130 °C for 20 min. The required area can then be cut and separated.

Figure 3d shows the separation of the PDMS mold to obtain a microchannel array with the transferred fiber pattern. Since the surface of the silicon wafer on which the fibers are placed is coated with a hydrophobic coating, the fiber mold base remains attached to the silicon wafer even after the PDMS is separated. In Figure 3e, the tunnel mold formed from PDMS is assembled with a glass slide to form a channel through which fluid can flow. For surface treatment, a UV–ozone cleaner was applied for 1 min; this is a dry, non-destructive, and chemical-free process that removes organic contaminants from the surface and efficiently converts hydrophobicity to hydrophilicity [26,27]. The surface-improved channel enhances capillary flow, which is described in detail in Section 3. The mold base can be reused, which is a repetition of (c) and (d) using the same fibers remaining on the silicon wafer after PDMS separation for PDMS replication. The results of reuse are presented in Section 3.2. In Figure 3f, applying an aqueous ink to the end of the channel causes fluid to be spontaneously drawn in and move along the channel due to capillary forces. In Figure 3g, the functionality of the fabricated channel is expanded by dynamically controlling the fluid velocity through photothermal effects. This application demonstrates that active flow control is possible even when capillary forces are insufficient due to the hydrophilicity and size of the channel interior. Therefore, this study presents a method for fabricating microchannels for flow control very simply and rapidly.

## 3. Results and Discussion

### 3.1. Polymeric Fibers

Let us examine the NFES step in detail. To eliminate potential adverse effects caused by variations in the polymeric fiber’s travel distance, the TTCD was fixed at 0.6 mm, and the applied voltage was adjusted in increments of 0.1 or 0.2 kV within the range of 1.1–1.9 kV. Among these, conditions of 1.4, 1.5, 1.7, and 1.9 kV were applied for channel width comparison. Additionally, the experiment was conducted in an environment with a temperature of 78–80 °F and a relative humidity of approximately 20% [19]. Through the programmed movement of the stage, the position of the fiber can be precisely controlled according to the resolution of the moving stage.

**Figure 4:**
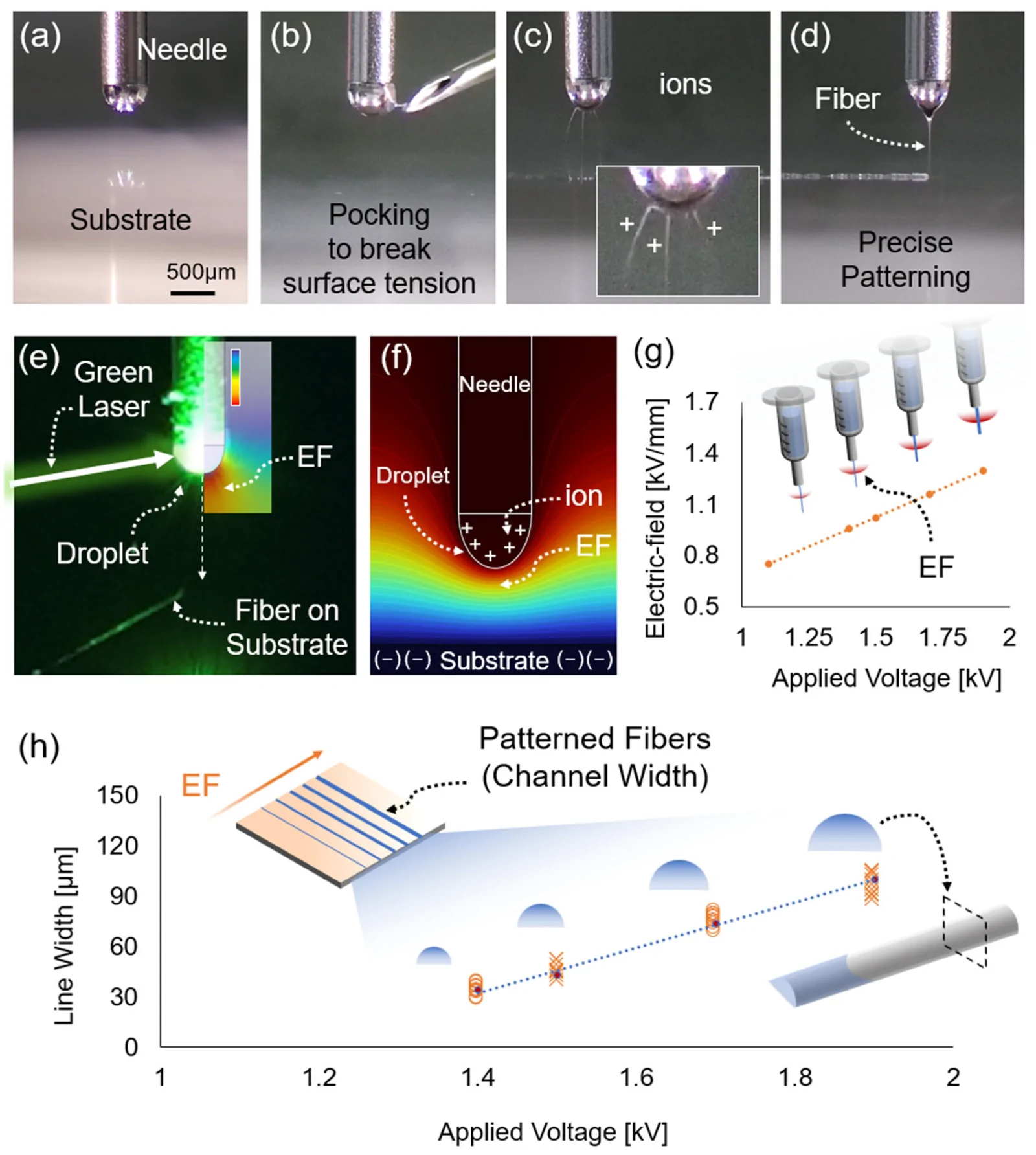
Near-field electrospinning steps and fabricated fibers. (**a**) Setting up the needle, substrate, and polymer solution. (**b**) Poking the droplet to break surface tension of the droplet. (**c**) The fiber jets on the droplet are locally generated and the fibers are ejected by the electric field. (**d**) Optimized electric field merges fibers and creates precise patterning. (**e**) Real-time monitoring of fiber ejection using a laser. (**f**) Electrostatic force distribution simulation supports the theory that the strongest electric field attracts the fiber. (**g**) As the voltage increases, the strength of the electric field increases linearly. (**h**) Higher applied voltage induces a stronger electric field, leading to an increase in the volume of the polymer fiber.

Figure 4a shows the preparation step for initiating NFES. Precise device setup for NFES improves fiber quality [28]. A stable TTCD is maintained in the near-field region, ensuring the electrohydrodynamic jet remains stable. Silicon wafers with high flatness help maintain a constant TTCD in NFES [29]. By maintaining a constant TTCD and adjusting the applied voltage, the EF for fiber ejection can be precisely controlled. Consequently, fibers of a consistent size can be manufactured during printing. In Figure 4b, a fiber jet is generated. As introduced in Section 2.2, the surface tension of the liquid droplet can be overcome by the Coulomb attraction between charges, allowing the fiber to be ejected. However, inducing a cone jet using only the force of an EF can cause the droplet to break or fall. Therefore, when the surface tension of the droplet and the EF are in balance, a needle is used to perform poking. Locally, the surface tension of the viscous droplet is broken, and the polymer is ejected by the EF. At this time, multiple fiber jets by repeated poking may be generated, as shown in Figure 4c [16]. In this case, since the fiber jets have the same (+) ions, they do not merge with each other but produce separated fiber jets. The direction of the fibers’ ejection is toward the silicon wafer containing (−) ions. This phenomenon demonstrates the theoretical principle that ionized optical fibers are attracted to the collector. In Figure 4d, the voltage gradually increased from 1.4 kV to find the optimal point where fibers merge and a single fiber is ejected. Once a stable fiber is ejected, the stage speed is adjusted to create a precise fiber pattern. The stage speed is set at 0.7 mm/s. At 1 mm/s, the stage speed is too fast, causing the fiber to break, while at 0.5 mm/s, the fiber ejection speed is higher than the stage speed, creating a coil-shaped pattern. The stage moves at a controlled speed to form parallel PEO fiber mold bases with a target width of approximately 34 to100 μm, as shown in the attached image, and the line spacing is adjusted according to the desired channel spacing.

Figure 4e further visualizes real-time monitoring of fiber ejection using a laser. A commercial green laser pointer (532 nm) is projected onto the droplet. The laser refracts and reflects inside the transparent droplet, causing it to emit light. This light propagates to the ejected fiber, making not only the fiber jet but also the fiber placed on the collector substrate visible. Thus, we can recognize that NFES can form neat lines rather than randomly collected nanofiber mats like those produced by conventional far-field electrospinning. Far-field electrospinning (FFES) uses a relatively large needle-to-collector distance and a strong electric field to stretch the polymer jet over a long flight path. During this process, the jet undergoes bending and whipping instabilities, causing the fibers to deposit in random directions and form a nonwoven, randomly oriented fiber mat [30]. In contrast, NFES uses a much shorter needle-to-collector distance and a controlled electric field. Because the jet has less time and space to develop severe bending instabilities, the fiber can be directly written onto the collector along a programmed trajectory, enabling the fabrication of well-defined, aligned, and patterned fiber structures [31]. The inset image in Figure 4f shows the electrostatic force distribution, revealing that the region with the highest EF density extends from the bottom of the droplet to the collector substrate. The strongest EF (EF= applied voltage/TTCD) is expected to occur at the lowest point of the droplet. The fiber jet starts at the location where the strongest electrostatic force occurs and travels along a vertical path along the shortest TTCD. This simulation supports the theory that the strongest electric field attracts the fiber along this path.

Figure 4g shows the simulated EF. As the voltage increases, the strength of the EF increases linearly from 0.75 to 1.3 kV/mm. According to the results of a study previously conducted by our research team, increasing the applied voltage increases the electric field strength between the nozzle and the collector, thereby increasing the volume of the fiber jet deposited on the substrate. However, even when the fiber discharge volume changes, the semi-elliptical cross-section formed on the substrate and the ratio of width to height were maintained. Therefore, the applied voltage can be used as a variable to control the width and height of the fiber [11]. Consequently, this implies that the size of the microfluidic channel can be controlled through the EF. The cross-section of the fiber placed is semi-elliptical, which is explained in the next section.

Figure 4h shows the width of the fabricated fiber according to the applied voltage. The average fiber width was fabricated to be 34 µm when the applied voltage was 1.4 kV, 43 µm when it was 1.5 kV, 74 µm when it was 1.7 kV, and 100 µm when it was 1.9 kV. However, a single fiber jet was not formed at 1.1 kV. The array marked with an “O and X” represents data obtained by measuring 10 different widths under each voltage condition. The width of the replicated PDMS channel was measured at 10 different locations along the linear channel section under each voltage condition, and the mean and standard deviation were determined. Under conditions of 1.4, 1.5, 1.7, and 1.9 kV, the average widths of the PDMS channel were measured to be 32.15 ± 3.46, 44.61 ± 3.75, 73.92 ± 3.91, and 95.87 ± 6.55 μm, respectively. As the applied voltage increased, the replicated PDMS channel width consistently increased, which supports the fact that the final channel width can be controlled through electric field control in the NFES process [32,33,34].

In Table 2, to compare the width-based dimensional matching between the fiber template and the replicated PDMS channel, the relative deviation between the representative fiber width and the average width of the PDMS channel for each voltage condition was measured. At 1.4, 1.5, 1.7, and 1.9 kV, the relative deviations were 5.44%, 3.74%, 0.11%, and 4.13%, respectively, and the average relative deviation for the four conditions was 3.36%. Therefore, under each voltage condition, the width of the fiber template and the average width of the replicated PDMS channel maintained similar values, and it can be argued that the NFES fiber geometry is reproduced in the PDMS channel based on width.

### 3.2. Fibrous Mold Base

**Figure 5:**
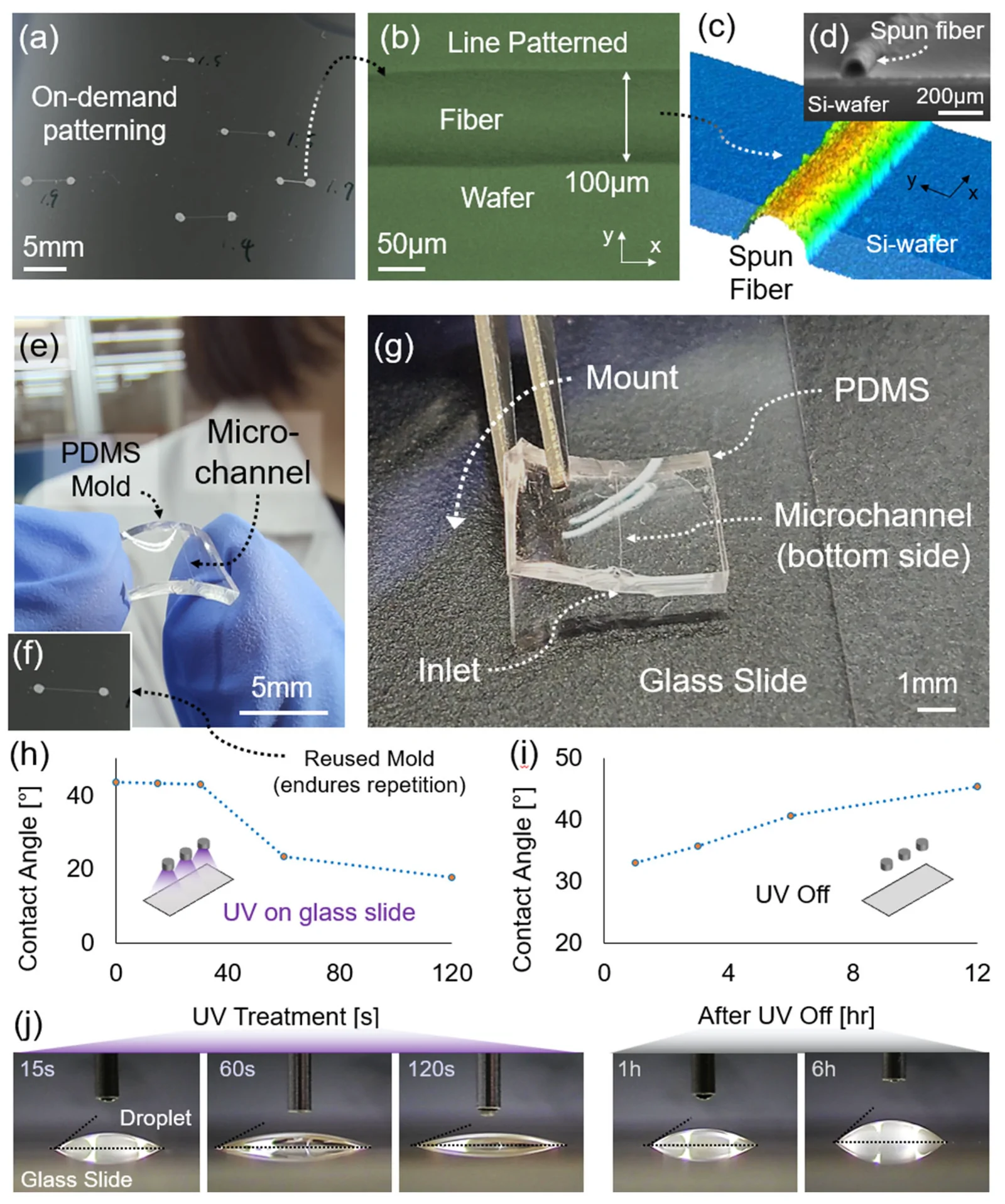
Fabricated mold base. (**a**) Straight and dot patterns for the on-demand position. (**b**) Microscopic view of the line pattern. (**c**) 3D scanned view of the patterned spun fiber that has semi-elliptical height. (**d**) SEM cross-sectional view of the spun fiber. (**e**) PDMS mold that replicates the shape of the fiber. (**f**) Reused fibrous mold. (**g**) Microchannels made of PDMS are attached to the glass slide facing each other. As a result, the fabrication of the channel device is completed. (**h**) Contact angle between the glass slide and water with UV exposure of 0, 15, 30, 60, and 120 s. (**i**) Surface recovery after 0.5, 1, 3, 6, and 12 h. (**j**) Change in hydrophilicity between the water droplet and the glass slide surface over time when UV is exposed and then turned off.

Figure 5 presents the fabricated mold-based microchannel. Figure 5a shows an on-demand printing of the straight fibers and dot patterns introduced in Figure 3a,b at desired locations. Since the fibers were generated with different EFs as in Figure 4h, the width of the patterns varies. The dot patterns at the ends of the lines serve as reservoirs for the inflow ink. In Figure 5b, the microscopic image confirms that the generated line pattern remains straight; the boundary between the fiber and the wafer is clearly distinguishable because of the contact angle. As shown in the 3D scan image in Figure 5c, the fiber height along the *z*-axis exhibits a semi-elliptical profile. Since the cross-sectional geometry of the fabricated microchannel follows that of the deposited PEO fiber, the channel shape can be estimated from the fiber width. Previous studies have reported that the semi-elliptical fiber height increases proportionally with fiber width, and that fiber width increases with electric field strength [11]. Accordingly, when uniform fibers are produced through the NFES process, the resulting microchannels also maintain an approximately constant width along their length and adopt a geometry similar to the template shown in Figure 4h. Figure 5d is a cross-sectional Scanning Electron Microscopy (SEM) image of a PEO fiber formed on the silicon wafer. The PEO fiber extruded by NFES is deposited on the substrate and then forms a solid shape as the solvent evaporates, forming a semi-elliptical cross-section corresponding to a semi-cylindrical surface profile.

Figure 5e shows the PDMS mold replicated from the fiber geometry. After fiber deposition on the wafer, the surface is treated with silane to render it hydrophobic, and PDMS is then poured over the fibers and wafer and subsequently cured. Figure 5f shows reusability of the mold base. Even after PDMS peeling, the PEO fibrous mold base reliably remained on the silicon wafer surface, and additional PDMS replication was performed using the same mold base. After additional replication, the channel width ranged from 72.15 to 78.66 μm, which is similar to the initial measurement of approximately 74 μm. The linear section width of the channel remained similar even after 10 additional PDMS replications. This result is attributed to the hydrophobic coating functioning between the fiber template and the PDMS, which enables PDMS peeling without mechanical friction. The fibrous mold base geometry is maintained throughout multiple channel fabrications, and repetitive PDMS replication of the same mold base is successfully achieved. The cured PDMS preserves the negative replica of the fiber with high fidelity and is assembled onto a glass slide, as shown in Figure 5g. UV–ozone treatment was applied before assembly to clean the surfaces and facilitate bonding. The PDMS microchannel was then attached to the glass substrate through hydrophilic adhesion, completing device fabrication.

As shown in Figure 5h, the contact angle between the glass slide and water was measured to quantitatively evaluate the change in surface hydrophilicity following UV–ozone treatment. The UV exposure durations were set to 0, 15, 30, 60, and 120 s. After completing UV exposure under each condition, water droplets were formed using the pump. The shape of the water droplets formed on the glass slide surface was captured with a high-magnification camera and analyzed using ImageJ (Version 1.54). The contact angle was measured based on the reference line of the specimen surface and the tangent of the water droplet boundary. The contact angle before treatment was 43.49°, and under treatment conditions of 15 and 30 s, it was 43.30° and 43.04°, respectively, showing no significant change. On the other hand, as the treatment time increased to 60 s, the contact angle decreased to 23.37°, and at 120 s, it decreased to 17.70°. In addition, as shown in Figure 5i, the recovery of surface hydrophilicity over time after UV–ozone treatment was evaluated. Based on the 60 s UV–ozone treatment conditions applied to the channel fabrication in this study, the boundary contact angle between the glass slide and water was measured after 0.5, 1, 3, 6, and 12 h in the air and normal temperature. The contact angle, which was 23.37° immediately after treatment, increased to 32.28°, 32.99°, 35.73°, 40.62°, and 45.43° after 0.5, 1, 3, 6, and 12 h, respectively. The contact angle recovered almost linearly over time, and after about 12 h, it returned to a state similar to that before UV treatment. Figure 5j shows water droplet images for UV-on and UV-off conditions, respectively, used for contact angle analysis. It demonstrates that hydrophilicity is enhanced with UV surface treatment, which implies that PDMS bonding to the glass surface is improved.

### 3.3. Capillary Force-Driven Flow

This section theoretically analyzes the movement of the liquid interface due to capillary driving and examines the change in flow velocity according to channel width in conjunction with numerical analysis results. For the initial flow, the initial flow velocity according to channel dimensions is calculated using the relationship between capillary pressure and the Bernoulli equation, and during the filling process inside the channel, capillary flow characteristics are analyzed by considering both inertia and viscous resistance. Additionally, we demonstrate flow differences at different channel widths through numerical analysis. This process mimics the phenomenon of fluid movement without an external pump in a fabricated PDMS–glass channel in the capillary flow experiments that follow in the next section. Therefore, we suggest that the process of theoretical calculations, numerical analysis, and experiments can be utilized complementarily in the design and fabrication of capillaries [35].

**Figure 6:**
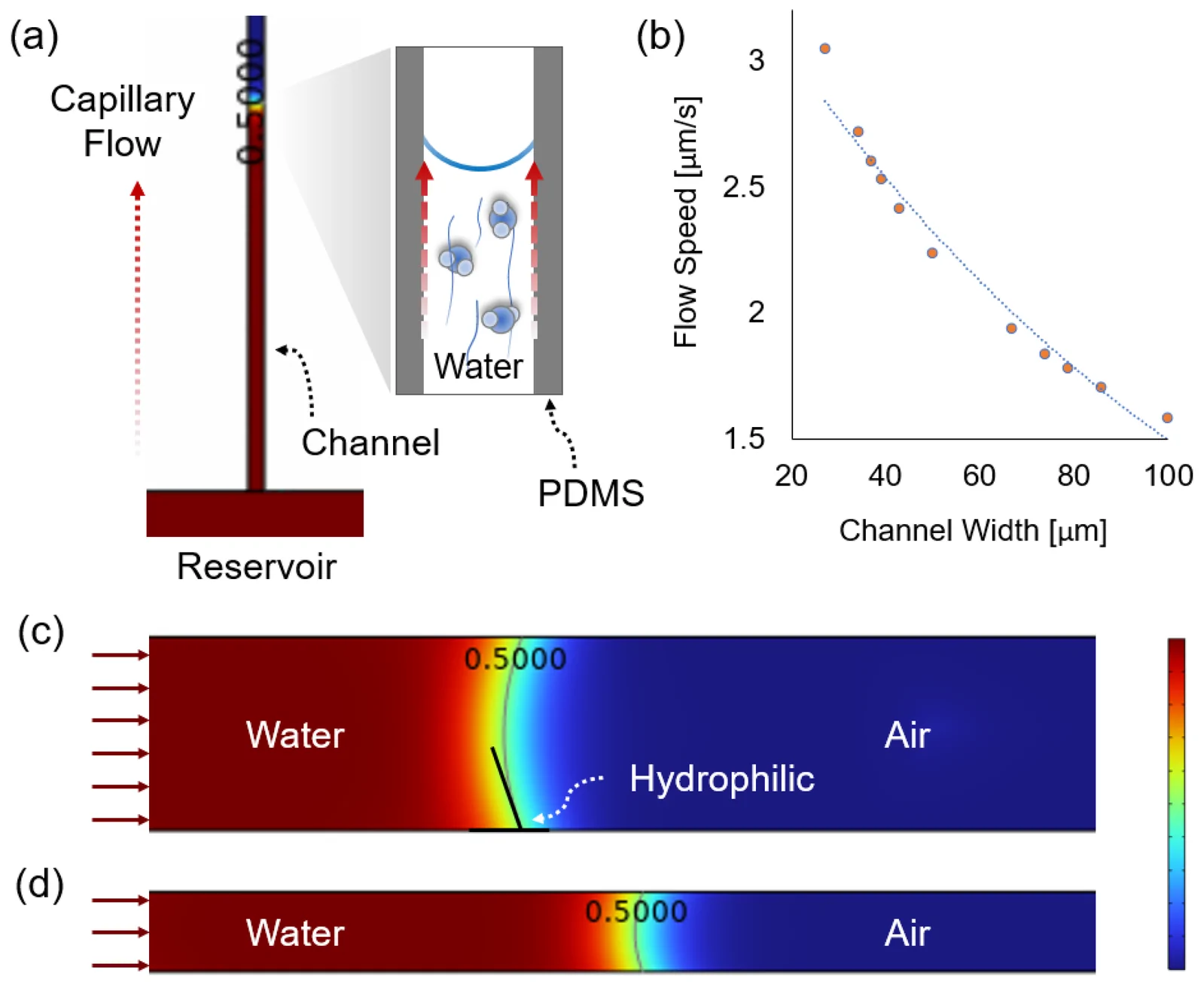
Flow simulation. (**a**) Capillary-driven water transport from an inlet reservoir into a microchannel. (**b**) Theoretical analysis confirms that capillary transport is significant in narrower channels. (**c**) Comparison of the flow behavior for different channel widths. (**d**) Simulated capillary forces can reliably drive fluid transport in microchannels, but the resulting flow velocity is highly sensitive to channel dimensions.

Figure 6a illustrates capillary-driven water transport from an inlet reservoir into a microchannel [36]. As the liquid penetrates the confined channel, its motion is governed by the balance among capillary pressure, viscous resistance, and interfacial forces at the liquid–air interface. Because the channel walls are hydrophilic, the meniscus advances spontaneously without external pumping, reflecting passive wetting-driven transport. This behavior is characteristic of passive microfluidic systems in which surface tension serves as the primary driving force. To theoretically understand the change in flow velocity according to channel width, the relationship between capillary pressure and the Bernoulli equation can be used [35,37].

(1) $$p_{1}\;+\;\frac{1}{2}\;\rho {V_{1}}^{2}\;+\;\rho gh_{1}\;=\;p_{2}\;+\;\frac{1}{2}\;\rho {V_{2}}^{2}\;+\;\rho gh_{2}$$

Under initial flow conditions, the inlet reservoir is sufficiently larger than the fabricated microchannel, so the flow velocity V_1_ inside the reservoir is assumed to be zero. Additionally, the channel is positioned horizontally, so the heights h_1_ and h_2_ inside the inlet reservoir and the channel are equal. Accordingly, the Bernoulli equation can be expressed as follows:

(2) $$p_{1}-p_{2}=\frac{1}{2}\;\rho {V_{2}}^{2}$$

Meanwhile, the capillary pressure caused by the curvature of the liquid interface can be approximated as follows using the Young–Laplace relationship [38].

(3) $$∆p_{\mathrm{cap}}=\frac{2\sigma \cos\theta }{r}$$

Here, ∆p_cap_ is the capillary pressure, σ is the surface tension of water, θ is the contact angle between the water and the channel wall, and r is the representative channel dimension applied in the initial flow velocity calculation. Since the channel in this study has a semi-elliptical cross-section, the measured channel width is approximated and applied as the representative dimension representing the interfacial curvature. Assuming that the pressure loss due to viscosity in the initial flow phase is relatively small, the dynamic pressure in Equation (2) and the capillary pressure in Equation (3) can be expressed in equilibrium as follows.

(4) $$\frac{2\sigma \cos\theta }{r}=\frac{1}{2}\;\rho {V_{\mathrm{init}}}^{2}$$

Therefore, the initial flow rate generated by capillary pressure can be calculated as follows: [39]

(5) $$V=\sqrt{\frac{4\sigma \cos\theta }{\rho r}}$$

V_init_ is the theoretically calculated initial flow velocity, and ρ is the density of water. For the calculation, the surface tension of water σ = 0.072 N/m^2^, contact angle θ = 30°, and ρ = 1000 kg/m^3^ were applied. According to Equation (5), when the physical properties of water and channel surface conditions are the same, the capillary pressure and the calculated initial flow velocity increase as the representative channel dimensions decrease.

Figure 6b presents the theoretically analyzed relationship between channel width and flow speed. The calculation results showed that the initial flow velocity increased as the channel width decreased. Since small representative channel dimensions generate high capillary driving pressure, the channel dimensions act as a key shape variable determining the initial capillary flow velocity.

As the capillary flow continues, the distance traveled by the water increases, and the influence of viscous resistance also increases. Since the channel in this study has a semi-elliptical cross-section, the cross-sectional shape including the channel height, in addition to the channel width, was considered. Defining the channel width as ω and the channel height as h, the cross-sectional area A of the semi-elliptical channel was calculated as follows.

(6) $$A=\frac{\pi \omega h}{4}$$

To reflect the cross-sectional characteristics of the channel in the flow analysis, the hydraulic diameter D_h_ was defined as follows.

(7) $$D_{h}=\frac{4A}{P_{\omega }}$$

Here, P_ω_ is the total wet perimeter of the channel cross-section in contact with the liquid. The hydraulic diameter D_h_ reflects the semi-elliptical cross-sectional characteristics including the channel width ω and height h. Assuming a uniform effective contact angle θ across the entire wet perimeter, the capillary force acting on the liquid–air interface can be expressed as σ P_ω_ cos θ. From the force equilibrium between the capillary force and pressure, the capillary driving pressure was derived as follows.

(8) $$∆P_{\mathrm{cap}}=\frac{\sigma P_{\omega }\cos\theta }{A}=\frac{4\sigma \cos\theta }{D_{h}}$$

Equation (8) implies that the smaller the hydraulic diameter, the higher the capillary driving pressure formed. If the distance traveled by the meniscus is defined as x(t) and the average flow velocity as dx/dt, the viscous pressure loss inside the channel can be expressed as follows, taking into account the hydraulic diameter and cross-sectional shape.

(9) $$∆P_{\mathrm{vis}}=\frac{P_{o}}{2}\frac{\mu x}{{D_{h}}^{2}}\frac{\mathrm{dx}}{\mathrm{dt}}$$

Here, μ is the dynamic viscosity of the liquid, and P_0_ is the Poiseuille number determined by the channel cross-sectional shape. By applying the flow rate Q = A dx/dt, the hydrodynamic resistance R_hyd_ was defined as follows.

(10) $$R_{\mathrm{hyd}}=\frac{∆P_{\mathrm{vis}}}{Q}=\frac{P_{o}\mu x}{2A{D_{h}}^{2}}$$

By excluding the gravitational term in the horizontal channel and applying the momentum equilibrium between the capillary driving pressure, inertia, and viscous resistance, the following Bosanquet-type relationship can be derived.

(11) $$\rho \frac{d}{\mathrm{dt}}\left(x\frac{\mathrm{dx}}{\mathrm{dt}}\right)+\frac{P_{o}}{2}\frac{\mu x}{{D_{h}}^{2}}\frac{\mathrm{dx}}{\mathrm{dt}}=\frac{4\sigma \cos\theta }{D_{h}}$$

Equation (11) considers the inertial effect of the initial filling process and the viscous resistance due to the increase in filling length. As filling progresses and the region dominated by viscous resistance becomes less effective, the effect of the inertial term decreases, so the equation can be rearranged as follows.

(12) $$\;\frac{P_{o}}{2}\frac{\mu x}{{D_{h}}^{2}}\frac{\mathrm{dx}}{\mathrm{dt}}=\frac{4\sigma \cos\theta }{D_{h}}$$

The following capillary filling relationship was derived by integrating Equation (12) with respect to time.

(13) $$x^{2}=\frac{16\sigma D_{h}\cos\theta }{P_{o}\mu }t$$

Equation (13) is a Lucas–Washburn-type capillary filling relationship that reflects the cross-sectional characteristics of a semi-elliptical channel through hydraulic diameter D_h_ and Poiseuille number P_0_, and expresses the proportional relationship between the square of the filling distance and time. In a semi-elliptical channel, the cross-sectional shape composed of the channel width and height affects both the capillary driving pressure and viscous resistance, and the capillary flow characteristics vary depending on the channel dimensions. In the initial flow analysis, the capillary driving pressure and initial flow velocity increased as the representative channel dimensions decreased, and in the numerical analysis, relatively high flow velocities were calculated in narrow channels. Therefore, the channel dimensions are a key shape variable that determines the initial capillary driving and subsequent viscosity-dominated filling behavior.

Since this model includes the fluid surface tension σ, dynamic viscosity μ, density ρ, and contact angle θ with the channel surface as material property variables, the capillary filling behavior can be analyzed by applying these material property values even to different liquids. The experimental flow verification of this study was performed on water; for applications to other liquids, the viscosity, surface tension, and wetting characteristics with the channel surface of the liquid must be considered together.

Figure 6c,d compare the flow behavior for different channel widths. In Figure 6c, the flow velocity decreases as the channel cross-sectional dimension increases under identical capillary driving conditions, owing to the greater hydrodynamic resistance associated with larger channel dimensions. In contrast, Figure 6d shows that with a narrower channel width, the interfacial curvature and capillary driving pressure increase, resulting in a higher predicted flow velocity. These results indicate that channel width strongly affects fluid transport dynamics, with faster capillary filling observed in narrower channels.

Overall, the simulation results support that capillary force can reliably drive fluid transport in microchannels, but the resulting flow velocity is highly sensitive to channel dimensions. Narrow channels promote faster transport, whereas wider channels reduce velocity because of increased hydraulic resistance. In addition, the dimensional fidelity of the fabricated channels is expected to depend on process parameters such as fiber jet stability, substrate surface energy after silanization, solution’s viscosity, and the assembly state during the fabrication process. If the process becomes unstable, incomplete channels may form due to local constriction or partial collapse. Therefore, it is noted that it is essential that the fabricated fibers remain continuous and free of breakage.

### 3.4. Achievement of Experimental Flows

**Figure 7:**
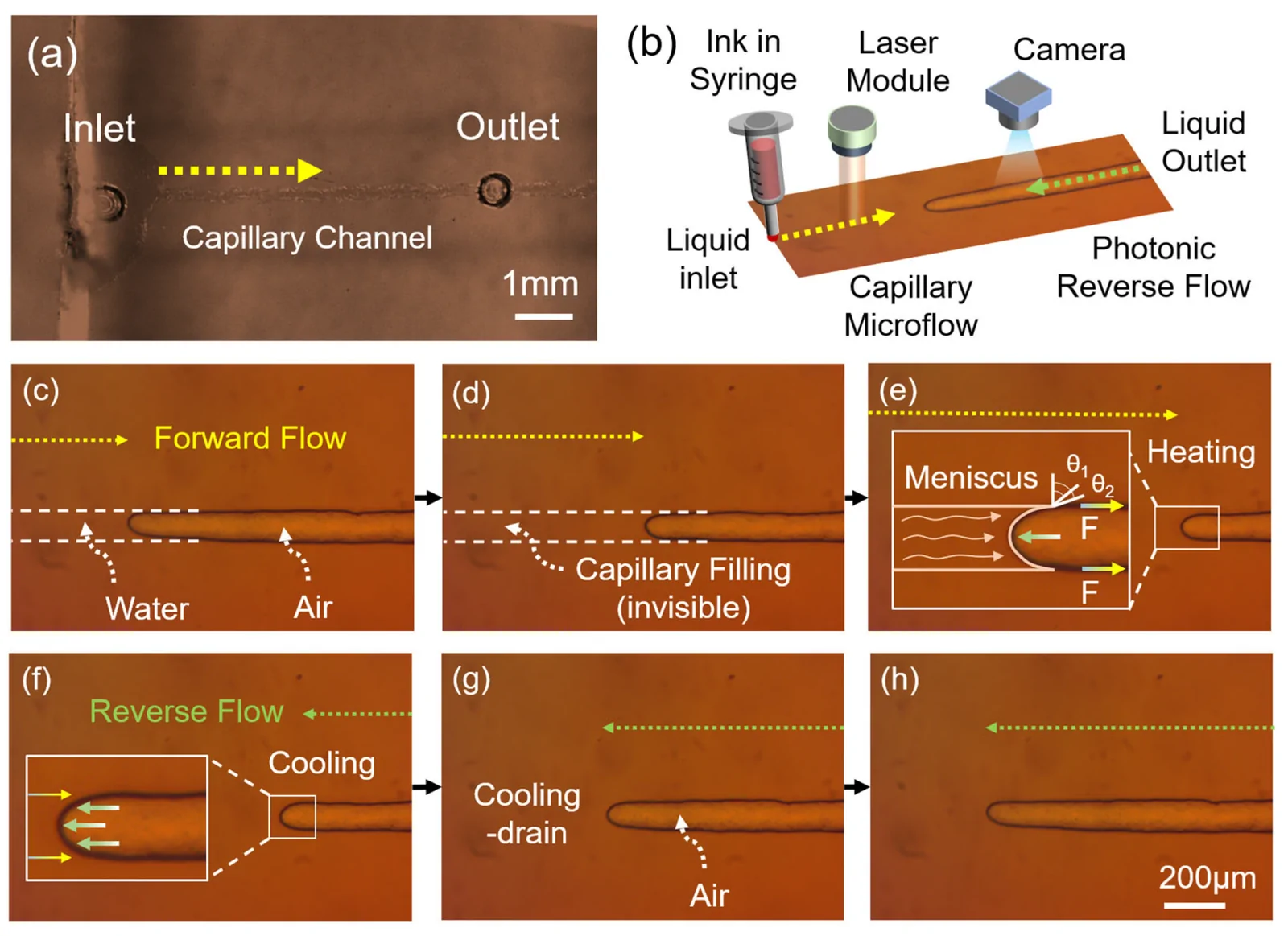
Demonstration of the implementation of fluid flow using the fabricated channel. (**a**) Transparent fluidic device assembled with a glass slide and PDMS made from NFES. (**b**) Conceptual figure for the experiments. (**c**) Water exhibits a natural flow from left to right. (**d**) As the fluid flows, it fills the channel. (**e**) Flow acceleration using photonic energy. (**f**) Reverse flow without light source (**g**) and (**h**) drain in cooling mode.

Figure 7 and Supplementary Video S1 present experiments demonstrating the implementation of fluid flow using the fabricated channel. Figure 7a shows a transparent fluidic device assembled with a glass slide and PDMS made from NFES. The circular pattern represents the reservoir, and the straight line in the center represents the capillary channel.

The schematic shown in Figure 7b essentially summarizes experiments on capillary microflow, natural flow, and light-assisted control. In Figure 7c, water is first injected into the built-in reservoir area of the channel created through the droplet-jetting process. Water exhibits a natural flow from left to right. As the fluid flows, it fills the channel as shown in Figure 7b, and this area becomes visually invisible. The refractive index of air is approximately one, but that of water is 1.33, which is similar to PDMS’s 1.4, causing the channel to become invisible [40]. Figure 7e demonstrates flow acceleration. When the inner walls of a 100 µm wide channel are not UV–ozone-cleaned, the capillary flow moves only about 3 mm. The issue is that sufficient actuation is not created between the channel and the water. In this case, forced flow is possible in two ways. First, pressure can be provided using an actuator. Second, the water can be heated up using light energy.

The practicality of this study is demonstrated by applying the second method to a fabricated channel. Photonic energy can improve flow velocity by simultaneously inducing a change in the water meniscus curvature, a reduction in the dynamic contact angle, and a decrease in viscosity. Capillary flow in a microchannel is determined by the balance between actuation pressure and viscous resistance. In hydrophilic channels, water typically forms a concave meniscus; as this curved surface forms, the component of surface tension (F) pulls the liquid toward the channel wall surface. Heating the water reduces surface tension, so the interface remains less “tight” at the same pressure difference, and as the curvature of the meniscus becomes gentler, the interface advances more easily [41]. The pressure difference in the capillary channel creates a flow, and this pressure difference is proportional to the contact angle cosθ [40]. When heated, the dynamic behavior of the interface changes, causing the contact line to move more easily, and consequently, the dynamic contact angle decreases from θ_1_ to θ_2_. In other words, cosθ increases and the flow velocity increases. Combined with the decrease in viscosity, the capillary flow velocity in the microfluidic channel increases. The effect of fluid temperature on capillary flow can be explained by applying temperature-dependent properties to the initial flow velocity relationship presented in Section 3.3. At temperature T, the initial flow velocity can be expressed as follows [42].

(14) $$V\left(T\right)=\sqrt{\frac{4\sigma \left(T\right)\cos\theta (T)}{\rho \left(T\right)r}}$$

σ(T), θ(T), and ρ(T) represent the surface tension, contact angle, and fluid density, respectively, depending on temperature, and r is the representative dimension of the channel. When light irradiation improves the wettability between the fluid and the channel wall, the contact angle θ(T) decreases, causing cos θ(T) to increase and the numerator of the equation to increase. Consequently, the capillary driving force acting on the meniscus increases, which can lead to an increase in the initial flow velocity. Furthermore, if the density ρ(T) decreases due to the thermal expansion of the fluid caused by the temperature rise, the denominator of the equation decreases, acting in a direction that increases the initial flow velocity. During the process of fluid moving into the channel, changes in dynamic viscosity μ(T) with temperature also affect the flow velocity. Viscosity-driven capillary flow can be expressed by the following relationship.

(15) $$\frac{\mathrm{dx}}{\mathrm{dt}}\propto \frac{\sigma \left(T\right)\cos\theta (T)}{\mu \left(T\right)x}$$

x is the distance traveled by the meniscus and μ(T) is the dynamic viscosity of the fluid. As the temperature rises and the dynamic viscosity decreases, the viscous resistance inside the channel decreases, thereby increasing the speed of the meniscus’s movement. Therefore, the additional advancement of the fluid observed after light irradiation can be explained as the result of the combined action of increased capillary driving force due to reduced contact angle, increased initial flow velocity due to reduced density, and reduced viscous resistance due to reduced viscosity [39].

Figure 7f–h show the process of reverse flow occurring when the photonic pumping was halted. When the light is stopped after the flow was forced by light energy to compensate for the insufficient capillary force, the meniscus surface moves back to 3 mm, which was the position where it stopped during natural flow, that is, it drains to the position where the driving pressure and viscous resistance are balanced when cooled. Therefore, the applicability of the idea for the facile fabrication technique presented in this study can be confirmed by utilizing capillary fluid movement and control application. The non-controllable light embedded in the microscope was 32 mW (measured with a SANWA LP10), and the surface temperature of the PDMS was around 50 degrees Celsius. Considering heat transfer within the PDMS channel and to the fluid, a multi-physics simulation integrating light and heat transfer would be required to accurately measure the amount of energy.

## 4. Conclusions

In this work, we demonstrated a facile microchannel fabrication strategy that uses NFES of PEO fibers as a mold base, followed by PDMS replication and simple assembly, to realize capillary microfluidic channels without the need for photomasks or conventional master molds. Conventional photolithography-based PDMS channel fabrication processes require the fabrication of separate photomasks and photolithography-based master molds for each new channel design. By leveraging direct-write NFES on a substrate, the process enables on-demand formation of straight lines and reservoir dots, silane-assisted demolding, and UV–ozone surface activation, resulting in rapid and reproducible fabrication of closed PDMS microchannels.

The proposed method allows for easy modification of the shape of the fiber-shaped mold base by changing the movement path of the programmed stage. We present that the dimensions of the final microchannel as well as the fiber-type mold base can be directly controlled through the adjustment of NFES process variables. Experimental study of the applied EF in the NFES process allowed us to tune the fiber width from 34 to 100 μm and consequently the channel dimensions, with the semi-elliptical profile of the deposited fibers faithfully transferred into the PDMS mold. This geometrical controllability, together with stable fiber jet formation and precise stage motion, ensured that channels with relatively uniform width and height could be produced, forming a reliable template for capillary-driven flow.

In the initial flow analysis, the capillary driving pressure and initial flow velocity increased as the representative channel dimensions decreased. Additionally, the capillary packing behavior was analyzed by reflecting the cross-sectional shape, including the width and height of the semi-elliptical channel as the hydraulic diameter, and considering inertia and viscous resistance together. Numerical analysis also supported the fact that narrower channels exhibited relatively higher velocities under the conditions of this study, indicating that channel dimensions are a key shape variable determining capillary flow. Experiments using aqueous solutions in the fabricated devices validated spontaneous, pump-free capillary flow and further showed that opto-thermal excitation can actively modulate flow by simultaneously altering meniscus, dynamic contact angle, and fluid viscosity.

Therefore, this study expanded the presentation of microchannel fabrication possibilities to demonstrate both pump-free passive capillary flow and the potential for active flow control using external stimuli. Thus, our main achievement is a facile fabrication process that goes beyond simple fiber patterning using NFES to the direct formation of a fiber-shaped mold base, PDMS replication, surface activation, glass substrate bonding, and the realization of capillary flow.

Overall, the proposed NFES-based fibrous mold-based approach provides a simple, low-cost, and facile route to microfluidic channels that can support both passive and light-assisted flow control, making it attractive for rapid prototyping and iterative optimization of capillary microfluidic platforms for applications such as biosensing, sample delivery, and lab-on-a-chip systems. Future work will focus on improving process robustness against jet instability and interfacial variations, extending the method to more complex channel geometries, and integrating additional functional elements for advanced microfluidic operations.

## Figures and Tables

**Table 1:** Comparison of channel fabrication methods.

Name	Method	Photomask/Separate Mold	Ease of Modification	Typical Size or Resolution	Advantages	Limitations
Photolithography	A master mold by photolithography, followed by replication	Required	New photomasks and master molds generally need	Generally at the micro-nanometer scale, depending on process conditions	High fabrication precision and excellent shape reproducibility	Requires a cleanroom, photomasks, and multiple fabrication steps
Sacrificial template	A removable form with internal channel geometry, followed by polymer formation and template removal	Generally unnecessary	Possible by modifying the template geometry	Varies depending on the template material and fabrication method	Enables the formation of enclosed and complex internal channels	Template removal is required, and residual material and channel deformation must be considered
3D printing	Channels from a digital model or a mold are produced for replication	Generally unnecessary	Relatively easy through modification of the digital model	Typically tens to hundreds of μm, depending on the printing method	Enables complex 3D structures and rapid modifications	Minimum feature size and surface quality depend on the printing method, equipment, and material
NFES in this study	Polymer fibers are directly deposited along desired paths and used as a mold base for PDMS replication	Not required	Easy by modifying the programmed path	Fiber diameters can range from nanometers to micrometers	Direct patterning of fine fibers, quick PDMS replication, reusable mold base	Geometric uniformity is affected by NFES process conditions

**Table 2:** Comparison of dimensions between fiber template and PDMS channel.

Applied Voltage [kV]	Fiber Template [µm]	PDMS Channel [µm]	Difference [µm]	Relative Deviation [%]
1.4	34	32.15 ± 3.46	1.85	5.44%
1.5	43	44.61 ± 3.75	1.61	3.74%
1.6	74	73.92 ± 3.91	0.08	0.11%
1.7	100	95.87 ± 6.55	4.13	4.13%

## Data Availability

The original contributions presented in this study are included in the article/Supplementary Materials. Further inquiries can be directed to the corresponding author.

## Supplementary Materials

The following supporting information can be downloaded at https://www.mdpi.com/article/10.3390/polym18192444/s1, Video S1: Experimental Capillary Flow in the Channel.

## Abbreviations

PDMS	Polydimethylsiloxane
FFL	Fused Filament Fabrication
VPP	Vat Photopolymerization
NFES	Near-field electrospinning
FFES	Far-field electrospinning
EF	Electric field
TTCD	Tip-to-collector distance
PEO	Polyethylene oxide
